# Swine as a potential reservoir of shiga toxin-producing Escherichia coli O157:H7 in Japan.

**DOI:** 10.3201/eid0506.990618

**Published:** 1999

**Authors:** M Nakazawa, M Akiba


### Swine as a Potential Reservoir of Shiga Toxin-Producing *Escherichia coli* O157:H7 in Japan

**To the Editor:** Shiga toxin-producing *Escherichia coli* (STEC) O157:H7 has become a major meat safety issue worldwide. Cattle, an important reservoir of human infection (1), may not be the only source of this organism (2,3). In a survey of pigs in England (4), non-STEC O157 was isolated from four (0.4%) fecal samples collected (after slaughter) from 1,000 pigs. We found that, although an unlikely source of infection for humans, pigs are a potential reservoir of STEC O157:H7 in Japan.

In 1997, there were 14,400 pig farms and 9,823,000 pigs (average 682 per farm) in Japan. Thirty-five (0.24%) of these farms were randomly selected for study, and rectal swabs were taken from 221 healthy pigs during May and June 1997. The average number of animals examined on each farm was 6.3.

Fecal samples were dipped into test tubes containing Cary-Blair transport medium (Nissui, Japan) and kept refrigerated until processing (usually within 48 hours). Swabs were then incubated overnight at 42°C in 10 ml of mEC broth (Kyokuto, Japan) containing 20 µg/ml of novobiocin (Sigma, USA), after which one loop of the broth was spread onto MacConkey sorbitol agar medium (Difco, USA). After overnight incubation at 37°C, sorbitol-negative colonies from the agar plates were tested by slide agglutination with *E. coli* O157-latex test (Oxoid, UK). Strains that agglutinated were confirmed as *E. coli* by using the API 20E system (BioMerieux, France). Strains confirmed as *E. coli* O157 were subcultured in a motility medium for 3 to 4 days to enhance development of flagella, then they were tested by tube agglutination with *E. coli* H7 antiserum (Denka-seiken, Japan). The swine *E. coli* O157:H7 isolates were examined by polymerase chain reaction for the presence of Shiga-toxin genes *stx1* and *stx2* and to elucidate intimin (*eaeA*) DNA sequences (5), for a plasmid of 92 kb (pO157) by agarose gel electrophoresis (6), and for phage type by the previously described method (7).

Although the numbers sampled were too small to allow comparisons between farms, samples from three (1.4%) apparently healthy pigs (ages: 2, 6, and 9 months) from three farms (8.6%) were positive for STEC O157:H7. The three strains from the pigs were biochemically typical of STEC O157:H7 that did not ferment sorbitol and lacked ß-glucuronidase; agglutinated with *E. coli* O157-latex and with H7 antiserum; possessed *stx1*, *stx2*, and *eaeA* genes; and harbored pO157 plasmid characteristic of STEC O157:H7. The strains belonged to phage type 21, 37, or 43.

The 1.4% carriage rate of STEC O157:H7 in pigs in this investigation is almost the same as that in cattle in Japan (8), which suggests that STEC O157:H7 strains are probably widespread in Japanese pig populations. The STEC O157-positive pigs were each housed in a concrete-floored pen and kept separate from cattle. Whether these pig isolates are the same as cattle or human isolates needs to be clarified; however, they had the same biochemical and genetic markers as STEC O157:H7 isolated from cattle and humans (6,9). The phage type 21 that we found among pig isolates was also observed in bovine and human STEC O157:H7 isolates in Japan (7). These results suggest that common vehicles for dissemination of the organism may exist.

So far, pork has not been identified as a source of human STEC O157:H7 illness in industrialized countries, but our results indicate that eating pork, contact with pigs, and contamination with pig feces should be considered potential sources of this pathogen. This is the first isolation of naturally occurring STEC O157:H7 in pigs in Japan.

### Acknowledgment

This work was supported by a grant from the Ministry of Agriculture, Forestry, and Fisheries of Japan.

**Muneo Nakazawa, Masato Akiba, Toshiya Sameshima**
National Institute of Animal Health,
Tsukuba, Ibaraki, Japan

### References

1. Wells JG, Shipman LD, Greene KD, Sowers EG, Green JH, Cameron DN, et al. Isolation of *Escherichia coli* serotype O157:H7 and other shiga-like-toxin-producing *E. coli* from dairy cattle. J Clin Microbiol 1991;29:985-9.
2. Chapman PA, Siddons CA, Harkin MA. Sheep as a potential source of verocytotoxin-producing *Escherichia coli* O157. Vet Rec 1996;138:23-4.

3. Chalmers RM, Salmon RL, Willshaw GA, Cheasty T, Looker N, Davies I, et al. Vero-cytotoxin-producing *Escherichia coli* O157 in a farmer handling horses. Lancet 1997;349:1816.
4. Chapman PA, Siddons CA, Cerdan Malo AT, Harkin MA. A 1-year study of *Escherichia coli* O157 in cattle, sheep, pigs and poultry. Epidemiol Infect 1997;119:245-50.
5. Sueyoshi M, Fukui H, Tanaka S, Nakazawa M, Ito K. A new adherent form of an attaching and effacing *Escherichia coli* (*eaeA+,bfp-*) to the intestinal epithelial cells of chicks. J Vet Med Sci 1996;58:1145-7.
6. Chapman PA, Siddons CA, Wright DJ, Norman P, Fox J, Crick E. Cattle as a possible source of verocytotoxin-producing *Escherichia coli* O157 infections in man. Epidemiol Infect 1993;111:439-47.
7. Akiba M, Masuda T, Sameshima T, Katsuda K, Nakazawa M. Molecular typing of *Escherichia coli* O157:H7(H-) isolates from cattle in Japan. Epidemiol Infect 1999;122:337-41.
8. Sekiya J. *Escherichia coli* O157:H7 in livestock in Japan. Revue Scientifique et Technique Office International des Épizooties 1997;16:391-4.
9. Ratnam S. March SB, Ahmed R, Bezanson GS, Kasatiya S. Characterization of *Escherichia coli* serotype O157:H7. J Clin Microbiol 1988;26:2006-12.

### Hospitalizations for Rotavirus Gastroenteritis in Gipuzkoa (Basque Country), Spain

**To the Editor**: Rotavirus is the main cause of severe acute gastroenteritis among children both in developing and in industrialized countries. The incidence of rotavirus gastroenteritis in northern Europe is similar to or greater than the estimated incidence of the disease in the United States (1-3); however, little is known about the impact of rotavirus infection on health in southern Europe.

We examined the incidence of hospitalization for rotavirus gastroenteritis during 3 years (July 1993-June 1996) in Gipuzkoa (population 400,480, of whom 58,896 are <15 years of age). The presence of rotavirus antigen was prospectively investigated by enzyme immunoanalysis (IDEIA Rotavirus, Dako Diagnostics, UK) in stool samples from all patients <15 years of age in the study area for whom a microbiologic analysis was requested for acute gastroenteritis. Children hospitalized for rotavirus gastroenteritis were sought retrospectively through searching both the computerized records of microbiology laboratory and hospital medical records for the diagnoses 558.9 (other gastroenteritis and presumably noninfectious colitis) and 008.6-009.3 (enteritis due to specific viruses and presumably infectious intestinal disorders) (4). All children in this study lived in the study area, had been hospitalized for gastroenteritis, and had one stool sample positive for rotavirus in the first 5 days of hospitalization without another gastroenteritis agent detected in the stool.

One hundred fifty-two (82 male and 70 female) of 1,004 children <15 years of age with rotavirus gastroenterititis had been hospitalized for rotavirus infection. No deaths were recorded. Cases usually occurred in epidemic waves, with the highest incidence in January-March. An additional 133 children with rotavirus in stools had been hospitalized but were not included in this study because they had hospital-acquired infections (67 cases), were coinfected by another microorganism (11 cases), came from outside the geographic study area (19 cases), or had a main reason for hospitalization other than gastroenteritis (36 cases). The mean annual incidence of hospitalization was 0.86 per 1,000 children (1 month to 14 years old) and 3.11 per 1,000 children (1 month to 5 years old). The maximum incidence occurred in 6- to 11-month-old children (11.81 per 1,000 children). Children were hospitalized for a mean of 4.8 ± 2.2 days. Rotavirus gastroenteritis was responsible for 152 (2%) of 7,403 pediatric admissions. For the 1- to 35-month age group, community-acquired rotavirus gastroenteritis was responsible for 140 (4.6%) of 3,026 admissions.

Although the incidence is based solely on confirmed cases, the findings closely reflect disease incidence in our region. The National System of Health covers 100% of the reference population, and hospitalization of children in private institutions is rare. Stool cultures were taken for most children for gastroenteritis (94.5%), and the presence of rotavirus was investigated in every case.

The hospitalization rate observed in this study was similar to that reported in other studies from Sweden (2), Denmark (5), and the United States (6) and lower than that found in England and Wales (3). In Spain, reporting of rotavirus infection is not required, is not included in mortality registers, and is not the object of specific vigilance by sentinel surveillance systems. Therefore, information about the incidence and impact of rotavirus infection in Spain is scarce. However, two reports from Spain must be highlighted: one is based on a theoretical prediction using a statistical model (7) and the

